# Targeting VEGF/VEGFR to Modulate Antitumor Immunity

**DOI:** 10.3389/fimmu.2018.00978

**Published:** 2018-05-03

**Authors:** Ju Yang, Jing Yan, Baorui Liu

**Affiliations:** The Comprehensive Cancer Centre of Drum Tower Hospital, Medical School of Nanjing University, Clinical Cancer Institute of Nanjing University, Nanjing, China

**Keywords:** vascular endothelial growth factor, tumor, angiogenesis, immune, T cells

## Abstract

In addition to the crucial role in promoting the growth of tumor vessels, vascular endothelial growth factor (VEGF) is also immunosuppressive. VEGF can inhibit the function of T cells, increase the recruitment of regulatory T cells (Tregs) and myeloid-derived suppressor cells (MDSCs), and hinder the differentiation and activation of dendritic cells (DCs). Recent studies have investigated the role of antiangiogenic agents in antitumor immunity, especially in recent 3 years. Therefore, it is necessary to update the role of targeting VEGF/VEGFR in antitumor immunity. In this review, we focus on the latest clinical and preclinical findings on the modulatory role of antiangiogenic agents targeting VEGF/VEGFR in immune cells, including effector T cells, Tregs, MDSCs, DCs, tumor-associated macrophages, and mast cells. Our review will be potentially helpful for the development of combinations of angiogenesis inhibitors with immunological modulators.

## Introduction

Blood vessels are required for the growth and dissemination of a solid tumor. There are numerous growth factors involved in tumor angiogenesis, but foremost among them is the family of vascular endothelial growth factors (VEGFs). The VEGF family includes VEGFA, VEGFB, VEGFC, VEGFD, and placenta growth factor (PGF) (1). These ligands bind with different affinities to three endothelial receptor tyrosine kinases (RTKs), such as VEGFR1, VEGFR2, and VEGFR3, as well as co-receptors, including neuropilins and heparan sulfate proteoglycans (2, 3). VEGFA has been studied more than other family members and is a critical regulator of angiogenesis (4). VEGFA is usually referred to simply as VEGF. VEGFR1 binds to VEGFB and PGF and is a positive regulator of monocyte and macrophage migration (5). VEGFR2 is the main signaling VEGFR in blood vascular endothelial cells. The activation of VEGFR2 involves both canonical mediators (VEGF, processed VEGFC and VEGFD) and non-canonical mediators (Shear stress, gremlins, galectins, lactate, and LDL) (5–7). Both blood and lymphatic endothelial cells express VEGFR3 during early development and VEGFR3 is reintroduced into blood endothelial cells during angiogenesis during angiogenic sprouting in the retina (5, 8, 9). VEGFR signaling has been extensively studied by Simons et al. (5) and Sia et al (2).

Vascular endothelial growth factor promotes tumor angiogenesis through stimulating the proliferation and survival of endothelial cells and also by increasing the permeability of vessels and recruiting vascular precursor cells from the bone marrow (2). Unlike the formation of mature vessels under normal conditions, intratumor vessels are complex, disorganized, irregular, and leaky, resulting in hypoxia and the inefficient delivery of antineoplastic agents to the tumor microenvironment (10, 11). Besides, VEGF has some direct effects on cancer cells or cancer stem cells. VEGF might promote cancer cell proliferation through the activation of VEGFR1 signaling (12). A recent study indicated that VEGFA/neuropilin-1 pathway conferred cancer stemness *via* the activation of the Wnt/β-catenin axis in breast cancer cells (13). Zhao et al. found that VEGF promotes tumor-initiating cell self-renewal through VEGFR2/STAT3 signaling (14).

**Figure 1:**
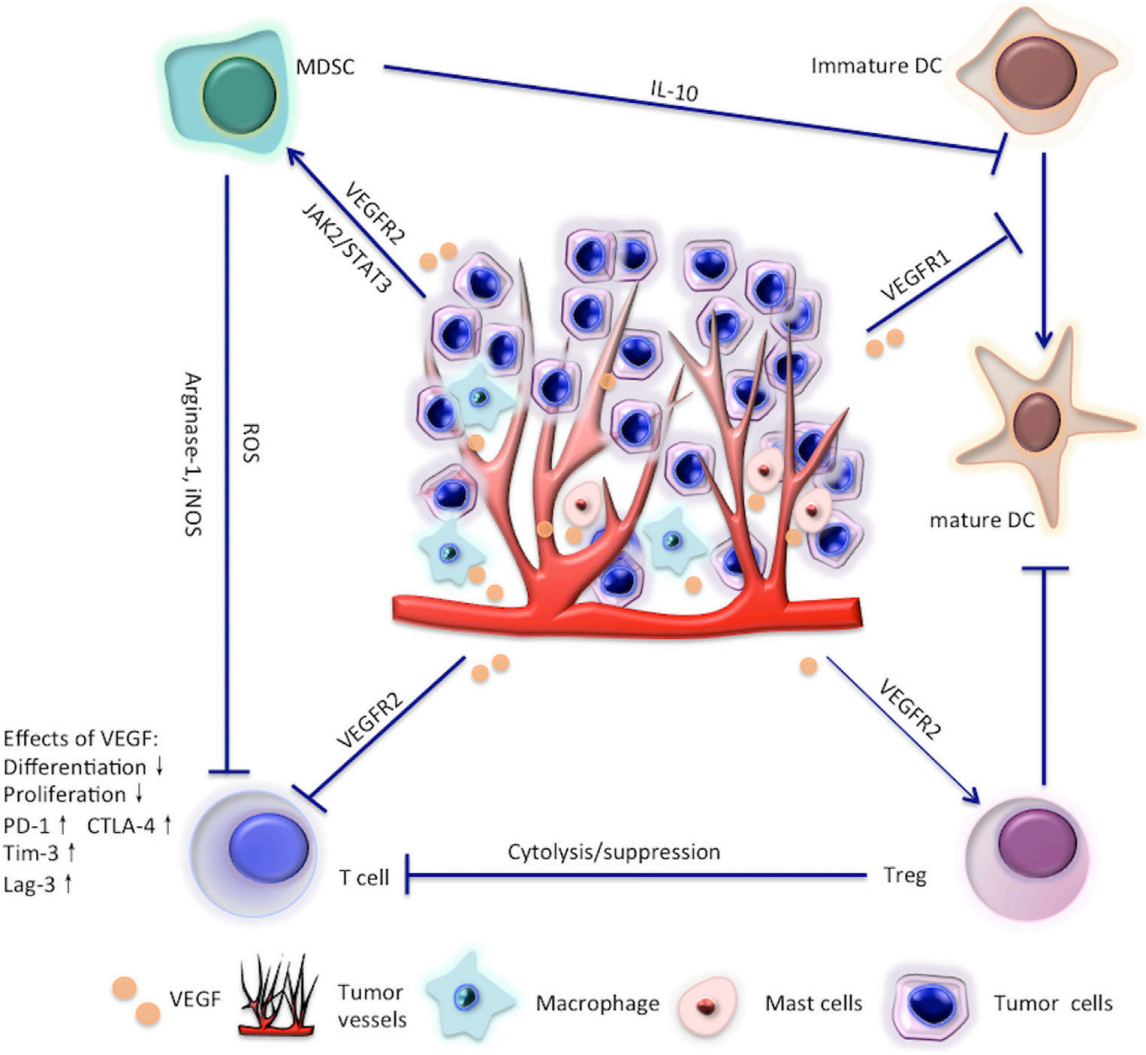
Effects of vascular endothelial growth factor (VEGF) on T cells, regulatory T cells (Tregs), myeloid-derived suppressor cells (MDSC), and dendritic cell (DC).

Meanwhile, VEGF is also immunosuppressive. The effects of VEGF on immune cells are summarized in Figure 1 and are reviewed in detail in the main text. Given the immunosuppressive role of VEGF, scientists have recently tried to restore the antitumor immunity by targeting VEGF/VEGFR. In this review, we focus on the latest clinical and preclinical findings on the modulatory role of antiangiogenic agents targeting VEGF/VEGFR in immune cells, such as effector T cells, regulatory T cells (Tregs), myeloid-derived suppressor cells (MDSCs), and dendritic cells (DCs).

## Effector T Cells

### Effects of VEGF on Effector T Cells

Zhang et al. found that strong expression of VEGF was detected in ovarian carcinoma tissues without T cells, while low expression of VEGF was detected in ovarian carcinoma tissues with T Cells (15). Ohm et al.’s study indicated VEGF impeded the development of T cells from early hematopoietic progenitor cells, indicating the potential immunosuppressive role of VEGF in tumors (16). But the direct effects of VEGF on T-cell function were not investigated in these two studies.

Basu et al. found that VEGFRs were expressed on recently activated and memory subsets of human CD4^+^ T cells (17). VEGF–VEGR interactions resulted in the activation of the MAPK and PI3K–Akt signaling pathways in human CD4^+^ T cells (17), similar to in endothelial cells (18). VEGF could also induce the production of IFN-γ and IL-2 and mediate migratory responses in human CD4^+^CD45RO^+^ memory T cells (17). However, mounting evidence supports the suppressive role of VEGF/VEGFR in T cells (19). Ziogas et al. found that VEGF significantly reduced the cytotoxic activity of T cells derived from peripheral blood samples, and that activated T cells expressed increased VEGFR2. Anti-VEGFR2 reversed the VEGF-induced suppression of T cells (20). Similar results were also observed in T cells from ascites secondary to ovarian cancer (21). In addition to the direct effects of VEGF on T cells, VEGF could also suppress T-cell function through combination with cyclooxygenase by upregulating FasL on the endothelium (22).

Kaur et al.’s study tried to illustrate the controversial role of VEGF in the activation of T cells. It was found that VEGF had context-dependent effects on T-cell activation. VEGF/VEGFR2 signaling inhibited TCR-dependent activation in T cells, but not in CD47-deficient T cells (23). VEGF and VEGFR2 expression were upregulated in CD47-deficient murine CD4^+^ T cells, and the resulting autocrine VEGFR2 signaling enhanced proliferation and some TCR responses in the absence of CD47 (23). This may explain the conflicting findings regarding whether VEGF was an inhibitor or stimulator in T-cell function. Because CD47 is ubiquitously expressed in human cells (24), it is possible that VEGF suppresses the function of T cells in most circumstances.

### Enhancing T-Cell Function by Antiangiogenic Agents Targeting VEGF/VEGFR

Regarding the immunosuppressive role of VEGF in T-cell function, it is biologically reasonable that interfering with VEGF/VEGFR can enhance antitumor immunity by improving T-cell function. There are some clinical and preclinical findings supportive of this hypothesis. Manzoni et al. found that bevacizumab (Avastin), a humanized anti-VEGF monoclonal antibody, could increase B-cell and T-cell compartments in patients treated with a bevacizumab-based first-line therapy for metastatic colorectal cancer (25). Bevacizumab also improved cytotoxic T-lymphocytes response in patients with metastatic non-small cell lung cancer (NSCLC) (26). Sunitinib is a multi-target tyrosine kinase inhibitor that can block VEGFR1, VEGFR 2, and VEGFR3, platelet-derived growth factor receptors α and β, stem cell factor receptor, and Flt3. Sunitinib was approved by the FDA for the treatment of renal cell carcinoma (RCC) and imatinib-resistant gastrointestinal stromal tumor (GIST) in 2006 (27, 28). Sunitinib was found to reduce the expression of IL-10, Foxp3, PD-1, CTLA4, and BRAF, but increased Th1 cytokine (IFN-γ) in isolated tumor-infiltrating lymphocytes (TILs) in an MCA26 (colon cancer cells) bearing mouse model. An increase in the proportion of CD4^+^ and CD8^+^ T cells was also observed in TILs in sunitinib-treated mice, whereas expressions of the inhibitory molecules PD-1 and CTLA4 were obviously reduced after sunitinib treatment. T cells from sunitinib-treated mice exhibited stronger cytotoxic activity against MCA26 tumor cells. These results indicate that sunitinib can modify the tumor microenvironment, resulting in a shift of cytokine and costimulatory molecule expression profiles that could favor T-cell activation and Th1 responses (29). Likewise, Schmittnaegel et al.’s study suggested that dual angiopoietin-2 and VEGFA inhibition elicited antitumor immunity by increasing the proportion of CD8^+^ T cells that expressed an activated, IFN-γ or CD69^+^ phenotype in both transgenic and transplanted mammary tumor models (30). Voron et al. found that VEGFA produced in the tumor microenvironment enhanced the expression of PD-1 and other inhibitory checkpoints involved in CD8^+^ T-cell exhaustion, including PD-1, CTLA-4, Tim-3, and Lag-3 (28). This effect could be reversed by antibodies targeting VEGFR2 (28), which is similar to Bamias et al.’s findings (21). Voron et al.’s study also indicated that VEGFA enhanced the expression of inhibitory checkpoints involved in T-cell exhaustion *via* the activation of the VEGFR2-PLCγ-calcineurin-NFAT pathway (28).

In addition to increasing T-cell activity, targeting VEGF/VEGFR also can promote T-cell infiltration in the tumor microenvironment. Targeting VEGF/VEGFR not only hinders the sprouting of new vessels (31, 32) but can also normalize the vasculature. Vasculature normalization can improve oxygen levels, drug delivery (33), and immune cell infiltration (34), especially in CD8^+^ T cells. This assumption is supported by abundant recent studies (30, 35–37). The extravasation of T cells into the tumor tissue depends on the expression levels and clustering patterns of intercellular adhesion molecule-1 (ICAM-1) and vascular cell adhesion molecule-1 (VCAM-1) (31, 38). VEGF can downregulate expressions or inhibit the clustering of these adhesion molecules to impair leukocyte–endothelial interactions (39–41). This can be reversed by VEGF antibody or inhibitor (30, 31, 34). There are some studies supporting the expression-promoting role of VEGF in adhesion molecules including VCAM-1, but most of them are not based on tumor models (42–44).

High endothelial venules (45) may be another mechanism by which T-cell infiltration can be promoted by targeting VEGFR2. HEVs are located in all lymphoid organs except the spleen and specialized postcapillary venules with portals through which blood-borne lymphocytes enter into secondary lymphoid organs (36, 46, 47). Recent studies have suggested that various human tumors could develop areas of HEVs and their presence was associated with a decreased tumor size and improved patient outcome (48, 49). A recent study by Allen et al. demonstrated that a combination of anti-VEGFR2 and anti-PD-L1 antibodies could induce HEVs in murine models. These HEVs enhanced lymphocyte infiltration and activity through activation of lymphotoxin β receptor (LTβR) signaling, and eventually improved the treatment efficacy (36).

## Targeting VEGF/VEGFR to Decrease the Number of Tregs

Regulatory T cells are immunosuppressive and can suppress or downregulate induction and proliferation of effector T cells (50). Tregs express the biomarkers CD4, FOXP3, and CD25 (51). The expression of VEGF has been shown to be positively associated with intratumoral Tregs, which are prognostic markers for the poor outcomes of various malignancies (52–54). Suzuki et al. showed for the first time that VEGFR2 is selectively expressed by FOXP3 high but not FOXP3 low Tregs (55). Neuropilins acted as co-receptors, increasing the binding affinity of VEGF for VEGFRs (56, 57). Promoted VEGF signaling through conjunction with neuropilin-1 may enhance Treg activation and create a tolerogenic environment (57). It is therefore reasonable that targeting VEGFA/VEGFR can modulate antitumor immunity by interfering with inhibitory Tregs.

Sunitinib has been reported to reduce the number of Tregs in tumor-bearing mice and in patients with metastatic renal carcinoma (29, 58–60). Sunitinib could target various receptors as mentioned above, and these studies (29, 58–60) did not investigate through which receptor sunitinib decreased the number of Tregs or the direct effects of VEGF on Tregs. Then, Terme et al. investigated patients receiving bevacizumab, a monoclonal antibody targeting VEGF, for metastatic colorectal cancer and treated colon cancer-bearing mice (CT26) with drugs targeting the VEGF/VEGFR axis. This study suggested that VEGF could promote the proliferation of Tregs and VEGF/VEGFR antibodies or inhibitors could decrease the number of Tregs in both patients with mCRC and the mouse models. This proliferation was inhibited by VEGF/VEGFR2 blockade (61), similar to the findings of Suzuki et al (55). In addition, sunitinib (55, 61), the anti-VEGFR2 antibody DC101 (62) and a chimeric receptor blocking VEGFR1/R2 (63) could also reduce the number of Tregs in tumors.

## Targeting VEGF/VEGFR to Inhibit the Accumulation and the Activity of MDSCs

Myeloid-derived suppressor cells were initially identified in tumor-bearing mice as cells co-expressing CD11b and Gr1 (64). Two main MDSC populations were characterized: monocytic MDSCs (M-MDSC) and polymorphonuclear MDSCs (PMN-MDSC). In tumor-bearing mice, PMN-MDSCs are the dominant population of MDSCs, while M-MDSCs are the dominant population for suppressing T-cell activation *in vitro* in human studies (64, 65). The mechanisms by which MDSCs elicited immunosuppressive effects can be grouped into four classes: lymphocyte nutrient depletion; generation of oxidative stress; interfering with lymphocyte trafficking and viability; and the activation and expansion of Tregs (64). Gabrilovich et al.’s study suggested that infusion of VEGF could increase the production of Gr1^+^ cells in tumor-free animals (66). The accumulation of MDSCs was shown to be associated with an increase in intratumoral VEGF concentration during disease progression in pancreatic-ductal adenocarcinoma-bearing mice (45).

Huang et al. found that VEGF could induce the accumulation of Gr1^+^CD11b^+^ cells by VEGFR2 and activation of JAK2 and STAT3 (67), but not VEGFR1 in tumor-bearing mice (68). Besides, MDSC enhanced by VEGF could induce the development of other immunosuppressive cells, including FOXP3^+^ Tregs, through a TGFβ-dependent and/or independent pathway (69–71). It is, therefore, reasonable to decrease the accumulation of MDSC by interfering with VEGF/VEGFR axis.

A decrease in the absolute number of MDSC in the spleen, bone marrow, and tumor in various tumor models has been observed after treatment with sunitinib (29, 58). The potential mechanisms included the following: sunitinib could act on MDSC by inhibiting STAT3; sunitinib could constrain the M-MDSC and lead to the apoptosis of granulocytic MDSCs (29, 58). Sunitinib also resulted in a favorable microenvironment depleted of MDSCs and synergize with HPV vaccine leading to enhanced levels of active tumor-antigen specific CTLs in a tumor-bearing mice model (72). Not only the quantity of MDSC but also the suppressive capacity was affected. In a melanoma-bearing mouse model, Axitinib, a small molecule against VEGFR1, R2, and R3, could induce a reduced suppressive capacity of MDSCs isolated from the spleen or tumor of Axitinib-treated mice compared to vehicle-treated mice. Moreover, treatment with Axitinib induced differentiation of MDSC toward an antigen-presenting phenotype (73). Clinically, sunitinib could result in a reduction of MDSC in RCC patients. The reduction of MDSC was correlated with reversal of T-cell suppression (74). A recent study demonstrated that bevacizumab-containing regimens significantly reduce the percentage of the granulocytic-MDSCs compared with non-bevacizumab-based regimens in patients with unresectable NSCLC (75).

## Dendritic Cells

### Effects of VEGF on the Differentiation, Maturation, and Activation of DCs

Dendritic cells are antigen-presenting cells of the immune system, which act as messengers between the innate and the adaptive immune systems. Immature DCs are derived from hematopoietic bone-marrow progenitor cells. Immature DCs are highly endocytic. They express relatively low levels of surface MHC-I, MHC-II, and costimulatory molecules such as CD80 and CD68. Hence, immature DCs are unable to process and present them efficiently to T cells (76, 77). Mature DCs are characterized by an increased capacity for antigen processing, increased the half-life of surface MHC-peptide complexes, and reduced antigen uptake (76, 78). Activated DCs can be distinguished from resting, mature DCs by expression of higher levels of MHC and costimulatory molecules or production of cytokines. Maturation and activation can occur simultaneously (76, 79). Thus, factors that interfere with the differentiation, maturation, and activation of DCs can lead to the dysfunction of DCs.

Clinical and preclinical studies indicated that VEGF could impair the differentiation and maturation of DCs. Almand et al. found that an increased plasma level of VEGF was associated with the presence of immature DCs in the peripheral blood of cancer patients. Surgical removal of the tumor could result in partial reversal of the observed effects (80). For patients with colorectal cancer, peripheral DCs were inversely correlated with VEGF serum levels (81). Various studies have indicated that VEGF binding to VEGFR1 blocked the activation of the transcriptional factor NF-κB and resulted in the inhibition of DC maturation in murine models (71, 82, 83). Dikov et al.’s study demonstrated that VEGFR2 affected the differentiation of DC from early hematopoietic progenitors (84).

## Targeting VEGF/VEGFR to Modify the Differentiation, Maturation, and Activation of DCs

Scientists have tried to modify the function of DCs by targeting VEGF/VEGFR. Sorafenib is a multikinase inhibitor and can inhibit RAF/MEK/ERK pathway, VEGFR2, VEGFR3, PDGFRβ, Flt-3, and c-KIT (85). Though various studies have investigated the associations between sorafenib and DCs, the results are inconsistent and the role of sorafenib in DCs remains controversial. Hipp et al. found that sorafenib impeded the maturation of DCs, characterized by reduced expression of CD1a, major histocompatibility complex, and costimulatory molecules in response to TLR ligands as well as by their impaired ability to migrate and stimulate T-cell response (85). However, Alfaro et al.’s study indicated that sorafenib could restore the differentiation of DCs assessed by the alloreactive mixed T-lymphocyte reaction (MLR) in the presence of VEGF and supernatants of RCC cells (86). The seemingly paradoxical results from the studies of Hipp (85) and Alfaro (86) are actually intelligible. Sorafenib does not target VEGFR1 and therefore will not stimulate DC maturation, while VEGFR2 is one of the targets of sorafenib and is responsible for the differentiation of DCs. A recent study by Zhao et al. suggested that sorafenib promoted the differentiation of bone marrow cells to immune suppressive DCs and constrained the MLR (87). In mouse models bearing hepatocellular carcinoma, Ho et al. found that sorafenib and TLR3 could enhance the activation of DCs (88). Discrepancies among different studies may lie in the intricate effects of sorafenib or distinct experimental designs, the complicated development of DCs, or the use of different animal models. Discrepancies also exist for sunitinib. Though sunitinib was found to increase the frequency of myeloid DCs in patients with renal cancer experiencing tumor regression (89), other studies indicated that the function of DCs was not affected by the use of sunitinib (85, 86). Generally, the exact roles of sorafenib and sunitinib in DCs are debatable and further studies are warranted.

A preclinical study indicated that bevacizumab could reverse the inhibitory effects of VEGF in the differentiation of monocytes into DCs *in vitro* (86). Various clinical studies suggested improvements in both quantity and function of DCs after the use of bevacizumab. Bevacizumab was found to increase the number of DCs in peripheral blood of cancer patients and enhance the allostimulatory capacity of DCs against recall antigens (90). In patients with metastatic NSCLC, bevacizumab was found to promote DC activation (26). Significant trafficking of CD163^+^ DCs across the tumor vasculature was observed in bevacizumab plus ipilimumab post-treatment biopsies in patients with metastatic melanoma, compared to ipilimumab alone (91).

## Tumor-Associated Macrophages (TAMs)

### Effects of VEGF on TAMs

Vascular endothelial growth factor can recruit macrophages to the tumor and promote TAM development (53, 92). TAMs have a poor antigen-presenting capacity and a decreased cytotoxic capacity due to the weak NO production (53, 93). TAMs can also hinder T-cell activation and proliferation by releasing IL-10, TGFβ, and prostaglandins (53, 94).

### TAM Might Be Involved in the Anti-VEGF Resistance

A recent study suggested macrophages could be actively recruited to the tumor microenvironment and were responsible for the anti-VEGF resistance in a mouse model bearing ovarian cancer. The resistance to anti-VEGF failed to occur in a macrophage-deficient mouse model (95). Zoledronic is a bisphosphonate drug and clinically approved for the treatment of bone metastases and osteoporosis. Bisphosphonates can also result in robust macrophage depletion (95–97). The addition of zoledronic acid at the emergence of resistance to anti-VEGF therapy halted tumor growth and obviously prolonged the survival of mice bearing ovarian cancer (95).

In addition to ovarian cancer, increased TAMs have been observed in the specimens of glioblastomas which progress during bevacizumab treatment and associated with a poor outcome in preclinical and clinical studies (98, 99). The accumulation of immune-suppressive cells was induced by increased expressions of stromal-derived factor (SDF)-1α (CXCL12) and C–X–C motif chemokine receptor 4 (CXCR4) (100, 101). SDF-1αpromoted the recruitment of macrophages by targeting CXCR4. Decreasing the recruitment of TAMs is another strategy to reverse the anti-VEGF resistance. A preclinical study by Deng et al. found that inhibition of SDF-1α inhibited the recruitment of TAMs induced by VEGF blockade and potentiated its antitumor efficacy in glioblastoma (102). Combination of VEGFR and CXCR4 inhibitors slows progression of GBM xenografts (100).

AMD3100 against CXCR4 was applied with a combination of bevacizumab in patients with recurrent high-grade glioma (HGG) in a phase I clinical trial NCT01339039. In ASCO annual meeting 2014, the preliminary data demonstrated that the combination treatment with bevacizumab and AMD3100 was well tolerated in HGG patients (103). Macrophage migration inhibitory factor (MIF) is another ligand for CXCR4. A recent study by Castro et al. described that MIF was also a mediator of increased macrophages and associated with bevacizumab-resistance in patients with glioblastoma and xenograft models by causing proliferative expansion of M2 macrophages (104).

## Antiangiogenic Therapy and Mast Cells

Kessler et al. found that mast cells accumulated in tumors before the onset of angiogenesis and resided in close proximity to blood vessels (105, 106). Mast cells can participate in the tumor rejection by producing molecules such as IL-1, IL-4, IL-6, and TNF-α. By contrast, mast cells can promote the tumor by enhancing its vascular supply, degradation of the tumor extracellular matrix and immunosuppression (107). Mast cells can synthesize and release angiogenic cytokines, including VEGF, FGF-2, the serine proteases tryptase and chymase, IL-8, TGFβ, TNF-α, and nerve growth factor (NGF) (107).

Similar to the findings in MDSC and TAM, antiangiogenic agents could reverse the tumor-induced immunosuppression by decreasing the tumor-promoting mast cells, and mast cells also played a role in antiangiogenic resistance. Axitinib is a multi-receptor inhibitor, which does not only inhibit VEGF receptors but also kinases including fms-like tyrosine kinase 3 (FLT-3), PDGF receptors, and CD117 (cKIT) (108). The latest study found that inhibition of mast cells by axitinib as well as their experimental depletion led to a decreased tumor growth. Treatment with axitinib also resulted in an improved T-cell response, which was pivotal for the therapeutic efficacy (109). A recent study demonstrated that mast cells could decrease the efficacy of antiangiogenic therapy (anti-VEGFR2 antibody DC101) (106). The potential mechanisms included: the degranulation-independent secretion of granzyme B, which liberates alternative pro-angiogenic factors including FGF-1 and GM-CSF from the ECM and the degranulation-dependent secretion of FGF-2 (106). Therefore, tumor-promoting mast cells might be a promising therapeutic target to improve the antitumor immunity and reverse the antiangiogenic resistance.

## Conclusion

In this review, we summarized the effects of VEGF and antiangiogenic agents on the immune cells, e.g., effector T cells, Tregs, MDSCs, DCs, TAMs, and mast cells. Agents targeting VEGF/VEGFR can restore the function and enhance the infiltration of effector T cells, decrease the number of immunosuppressive Tregs, TAMs, and mast cells, and inhibit the accumulation and immunosuppressive activity of MDSCs. But the effects of antiangiogenic agents on DCs are inconsistent among different studies and further studies are still needed. MDSC, TAMs, and mast cells also participate in the resistance of antiangiogenic therapy. Our review will be potentially helpful for the development of combinations of angiogenesis inhibitors with immunological modulators.

## Author Contributions

JY, JY, and BL conceived the theme of this review and wrote the manuscript.

## Conflict of Interest Statement

The authors declare that the research was conducted in the absence of any commercial or financial relationships that could be construed as a potential conflict of interest.
